# Identification of protein-damaging mutations in 10 swine taste receptors and 191 appetite-reward genes

**DOI:** 10.1186/s12864-016-2972-z

**Published:** 2016-08-26

**Authors:** Alex Clop, Abdoallah Sharaf, Anna Castelló, Sebastián Ramos-Onsins, Susanna Cirera, Anna Mercadé, Sophia Derdak, Sergi Beltran, Abe Huisman, Merete Fredholm, Pieter van As, Armand Sánchez

**Affiliations:** 1 Centre for Research in Agricultural Genomics (CRAG) CSIC-IRTA-UAB-UB, Campus UAB, 08193 Cerdanyola del Valles, Catalonia, Spain; 2 Faculty of agriculture, Ain Shams University, Khalifa El-Maamon st, Abbasiya sq, 11566 Cairo, Egypt; 3 Department of Veterinary Clinical and Animal Sciences, Faculty of Health and Medical Sciences, University of Copenhagen, Grønnegårdsvej 3, 1870 Frederiksberg, Denmark; 4 CNAG-CRG, Centre for Genomic Regulation (CRG), Barcelona Institute of Science and Technology (BIST), Baldiri i Reixac 4, 08028 Barcelona, Spain; 5 Universitat Pompeu Fabra (UPF), Barcelona, Spain; 6 Hypor, a Hendrix Genetics company, Spoorstraat 69, 5831 CK, Boxmeer, The Netherlands; 7 Hendrix Genetics Research & Technology Centre, Hendrix Genetics B.V, Spoorstraat 69, 5831 CK, Boxmeer, The Netherlands; 8 Departament de Ciència Animal i dels Aliments, Universitat Autònoma de Barcelona (UAB), 08193 Cerdanyola del Valles, Catalonia, Spain

**Keywords:** Taste receptors, Appetite-reward pathways, Coding genetic variation, Genotyping array, Future association studies

## Abstract

**Background:**

Taste receptors (TASRs) are essential for the body’s recognition of chemical compounds. In the tongue, TASRs sense the sweet and umami and the toxin-related bitter taste thus promoting a particular eating behaviour. Moreover, their relevance in other organs is now becoming evident. In the intestine, they regulate nutrient absorption and gut motility. Upon ligand binding, TASRs activate the appetite-reward circuitry to signal the nervous system and keep body homeostasis. With the aim to identify genetic variation in the swine TASRs and in the genes from the appetite and the reward pathways, we have sequenced the exons of 201 TASRs and appetite-reward genes from 304 pigs belonging to ten breeds, wild boars and to two phenotypically extreme groups from a F_2_ resource with data on growth and fat deposition.

**Results:**

We identified 2,766 coding variants 395 of which were predicted to have a strong impact on protein sequence and function. 334 variants were present in only one breed and at predicted alternative allele frequency (pAAF) ≥ 0.1. The Asian pigs and the wild boars showed the largest proportion of breed specific variants. We also compared the pAAF of the two F_2_ groups and found that variants in *TAS2R39* and *CD36* display significant differences suggesting that these genes could influence growth and fat deposition. We developed a 128-variant genotyping assay and confirmed 57 of these variants.

**Conclusions:**

We have identified thousands of variants affecting TASRs as well as genes involved in the appetite and the reward mechanisms. Some of these genes have been already associated to taste preferences, appetite or behaviour in humans and mouse. We have also detected indications of a potential relationship of some of these genes with growth and fat deposition, which could have been caused by changes in taste preferences, appetite or reward and ultimately impact on food intake. A genotyping array with 57 variants in 31 of these genes is now available for genotyping and start elucidating the impact of genetic variation in these genes on pig biology and breeding.

**Electronic supplementary material:**

The online version of this article (doi:10.1186/s12864-016-2972-z) contains supplementary material, which is available to authorized users.

## Background

There are five canonical tastes that are sensed in the taste buds of the tongue, which include salty, sour, sweet, umami and bitter. While salty and sour are detected by ion channels, the other three are sensed by a group of G-protein coupled receptors called taste receptors (TASRs). Sweet and umami are appetizing tastes that characterize energy-rich food sources, namely sugar and amino acid molecules, respectively, and are sensed by the TAS1R sub-type *TAS1R1*, *TAS1R2* and *TAS1R3* [1]. On the other hand, the – unpleasant - bitter taste indicating the presence of toxic molecules, is sensed by TAS2Rs, also known as bitter taste receptors [1], which include a variable list of highly polymorphic genes with many species-specific orthologs. The annotation of the pig genome contains ten TAS2Rs according to the Ensembl database (www.ensembl.org). In the recent years, it has become obvious that TASRs are expressed in many other tissues and have additional chemo-sensing functions. For example, they are present in the respiratory system where they regulate innate immunity and infection [2], and in sperm they have been linked to motility and acrosomal reaction [3]. In the gastro-intestinal tract, TASRs detect the molecules that are on transit and stimulate the appetite and reward (AR) circuitries to promote the appropriate feeding behaviour, thus keeping energy balance and body homeostasis [4, 5]. The AR mechanisms are highly interconnected and involve complex networks containing nutrients, neuropeptides, neurotransmitters, hormones and their related receptors and enzymes. These pathways engage the gastrointestinal tract, pancreas, liver, muscle, adipose tissue and brain. Appetite-related genes such as leptin (*LEP*), leptin receptor (*LEPR*), cholecystokinin (*CCK*), Ghrelin (*GHRL*), Agouti-related protein (*AgRP*), neuropeptide Y (*NPY*), proopiomelanocortin (*POMC*) and melanocortin 4 receptor (*MC4R*) encode for products that inhibit or excite the dopamine, epinephrine, norepinephrine, serotonin, and glutamate receptor pathways [6, 7] and modulate food intake and energy balance. In a nutshell, ghrelin and LEP are two hormones with opposite excitatory and inhibitory effects on the same neurons secreting appetite inducing NPY and AgRP or the feeding inhibitor POMC. These neuropeptides in turn, inactivate or excite MC4R, which is a hunger repressor. Ghrelin is secreted by the stomach when it is empty and LEP is released by adipocytes as a response to high energy stores [6]. CCK is a hormone secreted in the duodenum as a response to luminal fat and protein and is a strong inhibitor of food intake probably by decreasing gastric emptying and stimulating the vagus nerve [8]. LEP and ghrelin also inhibit and excite dopamine secretion, respectively [9]. Dopamine signalling in certain parts of the brain promotes appetite. Ghrelin secretion during fasting also promotes glutamate release. This neurotransmitter, via a large catalogue of receptors, will also excite NPY, AgRP and inhibit POMC neurons and boost appetite. The catabolic product of glutamate, gamma aminobutyric acid (GABA) also boosts appetite but using different neuronal mechanisms [10]. Moreover, glutamate is also able to excite dopamine-secreting neurons in appetite-relevant areas of the brain thereby indirectly promoting the feeling of hunger [9]. In contrast, GABA can inhibit the same neurons and indirectly promote satiety [9]. Serotonin, a neurotransmitter that is mostly present in the gastrointestinal tract but also at much lower levels in the central nervous system, modulates gastrointestinal motility, mood and appetite [11]. Serotonin inhibits appetite by stimulating its receptors HTR2C and HTR1B, which in turn, activate the well-known appetite-inhibitors POMC and MC4R and inhibit the appetite-promoter genes NPY and AGRP. Epinephrine/norepinephrine are two additional neurotransmitters that seem to be key in food intake and in keeping energy balance and have been shown to respond to starvation and low glucose levels in blood by activating the secretion of ghrelin [12].

In humans, the recent advent of whole genome [13] and exome [14] sequencing has shown that mutations severely impacting on protein sequence are more abundant than previously thought, although due to purifying selection, they tend to have very low allele frequencies. Thus, protein-damaging polymorphisms in TASRs and AR genes are likely to have an important impact on a broad range of traits including feed intake, immune function, behaviour or fertility both in livestock and humans. Consequently, understanding how these variants affect phenotypes in farm animals may both help improving the sustainability of the animal breeding sector as well as benefit bio-medical research. Scientific interest in this gene family in the pig is now emerging and it has recently been shown that TASRs are expressed in multiple porcine systems (immune, gastro intestinal, spermatogenic, etc. [15, 16]). The recent publication of the swine genome sequence and annotation [17] and the development of genome capture assays open unprecedented possibilities to identify deleterious genetic variation. For instance, 295 coding variants in swine TASRs have recently been identified after analyzing the low coverage whole genome sequences of 79 domestic and wild pigs from Europe and Asia [18].

Motivated by their functional relevance in the pig, we have sequenced ten porcine canonical TASRs and 191 AR genes in 304 pigs from multiple breeds in 16 DNA pools with the aim to identify coding polymorphisms and to provide a catalogue of potentially deleterious mutations likely to affect the function of these genes. Moreover, we have also detected indications that some of these variants might be associated with growth and fatness giving new insights in regard to the potential phenotypic relevance of polymorphisms within these genes.

## Results

### Sequencing statistics

We initially selected 459 kb of target genomic DNA (gDNA) covering the exons from the 12 TASRs and 201 AR genes. After genome capturing, sequencing and read mapping, we successfully covered 372 kb of target sequence with a read depth at each nucleotide position (DP) above 1,000 in the 16 libraries as a whole. This corresponds to 81 % of the initial target size and 201 genes fully or partially sequenced to a DP > 1,000. The poorly sequenced genes include *TAS2R3*, ENSSSCG000000029894 (a swine ortholog of human *TAS2R16*), and 10 AR genes. The list of successfully sequenced genes is shown in Additional file 1. After genome capture, sequencing and read mapping, 162,848,637 reads mapped to the target gDNA regions.

### Variant identification in TASR and AR genes

We successfully sequenced (DP ≥ 1,000) 14,598 bp (94.5 % of the initial selection) of TASR exons and identified 219 coding variants in TASR exons, 113 of which (52.1 %) do not have a dbSNP identifier and are thus considered novel (Additional file 2). Two TASR variants had the alternative allele fixed in the 16 pools and are thus likely to be either errors or private variants in the Duroc animal used to generate the reference sequence. We excluded them from our list of putative polymorphisms. Ten of the 217 remaining variants were classified by snpEff to have a high impact (H) on the coding sequence and consequently, on the function of the gene according to the gene annotation in the swine genome (Table 1). These include four single nucleotide variants (SNVs) and six short indels, which cause three stop-codon gains, one stop-codon loss, five frame-shift, and one novel splice-site donor. These variants affect four TASRs with a clear over-representation in *TAS1R1* (Table 1). Three of these variants affecting *TAS1R1* and *TAS1R3* had a predicted minor allele frequency (pMAF) ≤ 0.01 (Tables 2 and 3). In addition, we identified 125 non-synonymous-coding variants and one codon-deletion, which are classified by snpEff as having a moderate (M) impact. Thirty-four of the non-synonymous changes were predicted to be deleterious (Mdel) by SIFT [19] (Table 1). The remaining M variants were either SIFT predicted as tolerated (Mtol) or did not yield any prediction. Hence, we have identified 44 variants (10 H and 34 Mdel) that are likely to have an important effect on swine TASR function. Remarkably, all TASRs showed H or Mdel variants (Additional file 2). Finally, 81 variants were predicted to be synonymous changes with no apparent impact (L) on the subjacent proteins (Table 1). On average, the variants with strong impact on protein sequence (H and Mdel) were predicted to be rarer in the species than those having a mild impact (Mtol and L) (Table 2) according to pMAF.

**Table 1:** Number of variants across the TASR and AR gene groups per each impact class

	High impact					Moderate impact		Low impact			
Gene	Splice	Stop gained	Stop lost	Start loss	Frame shift	Mdel	Mtol/SIFT unknown	Silent	Start gained	% strong impact	Total
Total TASRs	1	3	1	0	5	34	92	80	1	20.3 %	217
TAS1R1 (umami)	1	1	1	0	3	4	9	12	0	32.2 %	31
TAS1R3 (sweet and umami)	0	1	0	0	0	1	0	11	1	14.3 %	14
TAS2Rs (bitter)	0	1	0	0	2	29	83	57	0	18.5 %	172
AR	37	16	0	4	17	277	615	1,559	24	14.5 %	2,549
Total segregating	38	19	1	4	22	311	707	1,639	25		2,766
Alternative allele fixed	4	0	0	0	4	0	9	9	1		27
Total	42	19	1	4	26	311	716	1,648	26		2,793

Splice: variants predicted to alter either donor or acceptor splice sites; % strong impact: percentage of H + Mdel variants

**Table 2:** Variant distribution per effect and pAAF within each gene group

	Variant frequency class	Strong impact	Mild impact
TASR	Very rare (pMAF < 0.01)	28 (63.6 %)	51 (29.5 %)
	Rare (pMAF = [0.010–0.019])	6 (13.6 %)	28 (16.2 %)
	Common (pMAF = [0.020–0.979])	10 (22.7 %)	94 (54.3 %)
	Total number	44	173
	pMAF min-max (average)	0.0016–0.1910 (0.028)	0.0016–0.4920 (0.091)
AR	Very rare (pMAF < 0.01)	184 (52.6 %)	560 (25.5 %)
	Rare (pMAF = [0.010-0.019])	41 (11.7 %)	235 (10.7 %)
	Common (pMAF = [0.020–0.979])	125 (35.7 %)	1,403 (63.8 %)
	Total number	350	2,198
	pMAF min-max (average)	0.0017–0.4800 (0.029)	0.0016–0.4980 (0.0992)

The percentages are for the total number of variants within the groups: TASR strong impact, TASR mild impact, AR strong impact and AR mild impact

**Table 3:** List of rare H variants

Variant ID	Effect	Gene	pMAF
chr12_15398873_C_T	STOP_GAINED	ACE	0.0017
chr12_62594159_A_G	START_LOST	ALDH3A2	0.0086
chr7_103136276_A_G	START_LOST	ALDH6A1	0.0017
chr7_103129739_C_G	SPLICE_SITE_ACCEPTOR	ALDH6A1	0.0034
chr9_110061864_G_A	STOP_GAINED	CD36	0.0017
chr7_28072405_A_C	SPLICE_SITE_DONOR	NOTCH4	0.0017
JH118674.1_43285_C_T	SPLICE_SITE_DONOR	GRIA1 ortholog (ENSSSCG00000024560)	0.0049
chr16_66618439_A_T	START_LOST	GABRG2	0.0091
chr16_66618438_C_A,T	START_LOST	GABRG2	0.0017
chr1_64002416_A_G	SPLICE_SITE_DONOR	GABRR1	0.0034
GL896494.1_7864_G_T	STOP_GAINED	GPR179	0.0052
chr13_203412188_T_A	SPLICE_SITE_ACCEPTOR	GRIK1	0.0058
chr1_77441762_G_A	SPLICE_SITE_ACCEPTOR	GRIK2	0.0034
chr6_49600987_C_T	SPLICE_SITE_DONOR	GRIN2D	0.0034
chr6_49600986_A_G	SPLICE_SITE_DONOR	GRIN2D	0.0034
chr13_37136736_G_T	SPLICE_SITE_ACCEPTOR	GRM2	0.0017
chr13_37136747_T_G	SPLICE_SITE_DONOR	GRM2	0.0034
chr7_34927184_A_C	SPLICE_SITE_DONOR	GRM4	0.0017
chr7_34893335_T_G	SPLICE_SITE_ACCEPTOR	GRM4	0.0017
chr18_3214922_AC_A	FRAME_SHIFT	HTR5A_human_ortholog (ENSSSCG00000030573)	0.0030
chr18_3151609_AG_A	FRAME_SHIFT	HTR5A_human_ortholog (ENSSSCG00000023549)	0.0020
chr3_55922763_C_T	SPLICE_SITE_ACCEPTOR	NPAS2	0.0020
chr1_14768986_T_G	SPLICE_SITE_ACCEPTOR	OPRM1	0.0017
chr5_85218678_C_A	STOP_GAINED	PAH ortholog (ENSSSCG00000000856)	0.0017
chr5_85218708_C_A	STOP_GAINED	PAH ortholog (ENSSSCG00000000856)	0.0017
chr5_85218747_G_T	SPLICE_SITE_DONOR	PAH ortholog (ENSSSCG00000000856)	0.0017
chr3_23446168_C_T	STOP_GAINED	SCNN1G	0.0017
chr16_85870363_G_C	SPLICE_SITE_DONOR	SLC6A3	0.0017
chr16_85870364_T_A	SPLICE_SITE_DONOR	SLC6A3	0.0017
chr6_58114941_G_A	STOP_GAINED	TAS1R3	0.0016
chr6_62357984_C_T	STOP_GAINED	TAS1R1	0.0065
chr6_62363203_G_C	STOP_LOST	TAS1R1	0.0065

The variant identifier (ID) contains information on chromosome _ position _ reference allele _ alternative allele

Likewise, we sequenced 357,844 (80.1 % of the initial selection) bp of exonic sequence from AR genes at a DP > 1,000, and identified 2,570 variant positions in AR exons, most of which were bi-allelic SNVs. 1,092 (42.4 %) variants were not annotated in dbSNP (Additional file 2). Four of these positions were multi-allelic with each allele predicted to have a different effect on protein sequence. Of the 2,574 assigned variant effects, 25 had the alternative allele fixed in the 16 pools. From the remaining 2,549 variants, 350 were classified as deleterious (H and Mdel) and affected 123 genes (Table 1 and Additional file 2). As for TASRs, H and Mdel as a whole tended to have lower pMAF than Mtol and L (Table 2). We identified 74 H variants in AR genes, 29 of which had pMAFs ≤ 0.01 and are thus considered rare. These H rare variants affected 21 genes (Table 3).

**Fig. 1:**
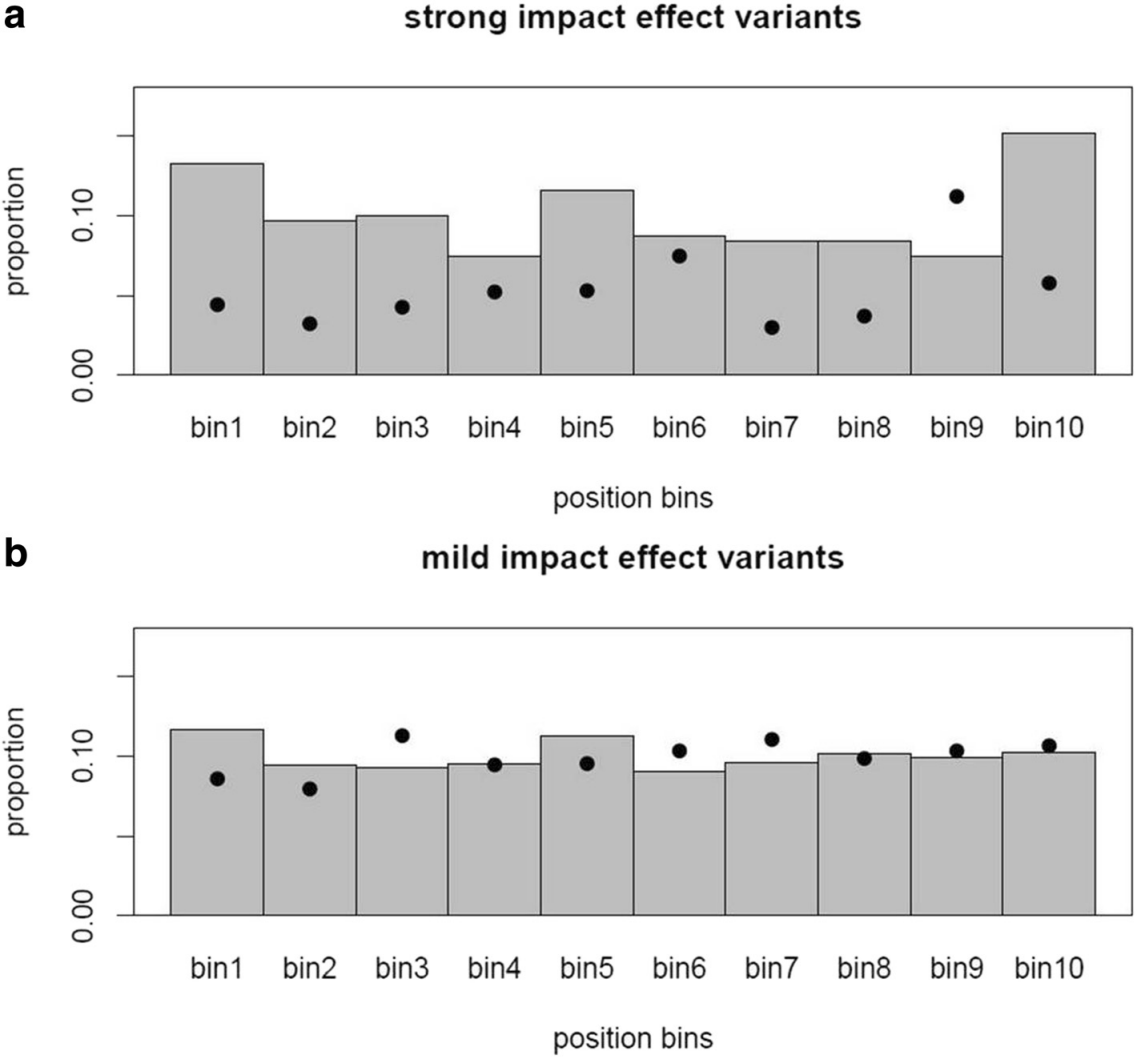
Distribution and pMAF of strong (H + Mdel) and mild (Mtol + L) impact variants along the protein body of AR genes. The bar plots for (**a**) the strong impact effect variants were made with 310 premature stop, frame-shift and Mdel. The barplot for the mild impact effect variants (**b**) were made with 2,174 Mtol, in-frame indels and synonymous variants. The dots indicate the average pMAF in each of the 10 position percentile bins. Percentile bins divide the protein body of each gene in ten portions of equal size

We also plotted the distribution of the AR gene variants predicted to have a strong impact on protein (H and Mdel) and those with a mild impact (Mtol and L) along the protein sequence divided in 10 consecutive position bins of equal amino acid length. We noted that the strong impact variants tended to be more abundant at the end of the protein (bin9 + bin10), and that these also tended to have, on average, larger pMAF. This trend was not observed in the set of mild impact variants (Fig. 1).

### Per breed variant distribution

We compared the ten purebred pools and observed that, as expected, larger pools contained more variants (both in TASR and AR genes). Nonetheless, the two Duroc pools together, with 45 samples, displayed less genetic diversity (1,042 variants), than the Large White, Landrace and the Pietrain, which had a similar number of animals and were above 1,300 variants each (Table 4). The Asian pool harboured the largest number of variants (1,589 variants with a pool size = 22) while the Iberian pool was ranked as the most homogenous (746 and pool size = 13). Overall, we observed that the most ancient breeds, Mangalitza, Iberian, Majorcan Black were less variable in our set of genes (Table 4). Given that a higher number of samples seem to be correlated to a higher number of variants, we also compared the genetic variability using the Watterson estimate [20] at the neutrally evolving synonymous sites (the third nucleotide position within each codon), which corrects the number of mutated sites by the number of animals included in the analysis. This value (Bazna: 0.00118, Mangalitza: 0.00101, Majorcan Black: 0.00103, Iberian: 0.00095, Duroc: 0.00090, Pietrain: 0.00131, Landrace: 0.00130, Large White: 0.00130, wild boar: 0.00109 and Asian: 0.00182) showed that all the populations had similar variability with the exception of the Asian pool, which genetic variability doubled that of the other breeds.

**Table 4:** Number of variants and number of unique variants within each breed and per impact class

	Duroc		Pietrain		Large white		Landrace		Bazna		Mangalitza		Iberian		Majorcan black		Wild boar		Asian	
Variant effect	nr (%)	nr unique	nr (%)	nr unique	nr (%)	nr unique	nr (%)	nr unique	nr (%)	nr unique	nr (%)	nr unique	nr (%)	nr unique	nr (%)	nr unique	nr (%)	nr unique	nr (%)	nr unique
Strong impact	133 (12.7)	1	144 (10.9)	1	140 (10.5)	1	124 (9.4)	0	99 (10.2)	2	87 (11.3)	4	92 (12.3)	2	90 (10.4)	1	123 (12.4)	6	163 (10.2)	26
High	44 (4.2)	1	46 (3.5)	0	49 (3.7)	0	33 (2.5)	0	28 (2.9)	0	29 (3.8)	1	30 (4.0)	1	30 (3.5)	0	35 (3.5)	0	48 (3.0)	9
Moderate_Deleterious	89 (8.5)	0	98 (7.4)	1	91 (6.8)	1	91 (6.9)	0	71 (7.3)	2	58 (7.5)	3	62 (8.3)	1	60 (6.9)	1	88 (8.9)	6	115 (7.2)	17
Mild impact	909 (87.3)	5	1177 (89.1)	5	1194 (89.5)	5	1195 (90.6)	3	868 (89.8)	11	685 (88.7)	8	654 (87.7)	9	778 (89.6)	21	870 (87.6)	32	1426 (89.7)	195
Moderate_Tolerated	279 (26.8)	4	342 (25.9)	1	332 (24.9)	2	342 (25.9)	1	257 (26.6)	2	192 (24.9)	2	187 (25.1)	5	223 (25.7)	9	252 (25.4)	15	389 (24.5)	50
Low	630 (60.5)	1	835 (63.2)	4	862 (64.6)	3	853 (64.7)	2	611 (63.2)	9	493 (63.8)	6	467 (62.6)	4	555 (63.9)	12	618 (62.2)	17	1037 (65.3)	145
Total	1042	6 (0.6 %)^a^	1321	6 (0.5 %)^a^	1334	6 (0.4 %)^a^	1319	3 (0.2 %)^a^	967	13 (1.3 %)^a^	772	12 (1.6 %)^a^	746	11 (1.5 %)^a^	868	22 (2.5 %)^a^	993	38 (3.8 %)^a^	1589	221 (13.9 %)^a^
Pool size	45		41		39		40		15		12		13		17		22		22	

This table was done using the 2,574 variants in TASR and AR genes including the multi-allelic with different effects and these with the alternative allele fixed in all the populations

^a^Percentage of variants that are breed-specific

We also compared the percentage of variants that were H or Mdel per breed. No obvious differences were observed (*p-val* = 0.07) and the Duroc and Landrace were at the top (12.7 %) and bottom (9.4 %) ends, respectively (Table 4).

Altogether, 26 TASR variants were present in a single breed at pAAF ≥ 0.1 and might thus be breed-specific. Not surprisingly, the Asian pool, which involved 15 Chinese Meishan and 7 Vietnamese pigs, showed the highest proportion of pool-specific variants, with 12 being present only in this group (Additional file 3). The wild boar also displayed several allelic particularities, with nine unique variants. Noteworthy, seven of these wild boar variants, all with similar pAAF, mapped to *TAS2R1* (Additional file 3). Overall, five (one H and four Mdel) breed-specific variants were predicted to have a strong impact on protein sequence (Additional file 3). The H variant is a stop gain in *TAS1R1* that is present in 17 % of the Mangalitza genomes, respectively. We also identified two variants, a synonymous (L) and a non-synonymous tolerated (Mtol) SNVs that affect *TAS2R1*, that whilst being present at very high frequencies or even fixed in all breeds (pAAF ≥ 0.5), were absent in the Asian pool (Additional file 3).

We detected 306 AR coding variants that were uniquely present or absent in one breed (Additional file 3). Of these, 35 variants involving 32 genes were predicted to be of functional importance (Additional file 3). As in TASRs, the breed specific H and Mdel variants in the AR genes were more abundant in the Asian and the wild boar with 20 and five unique features, respectively. It was noteworthy that two of these H and Mdel variants, both in the Asian pools, were close to fixation (pAAF ≥ 0.9). These variants map to the serotonin receptor, *HTR3C*, and to the cytochrome P450 gene, *CYP2A6* (Additional file 3).

**Fig. 2:**
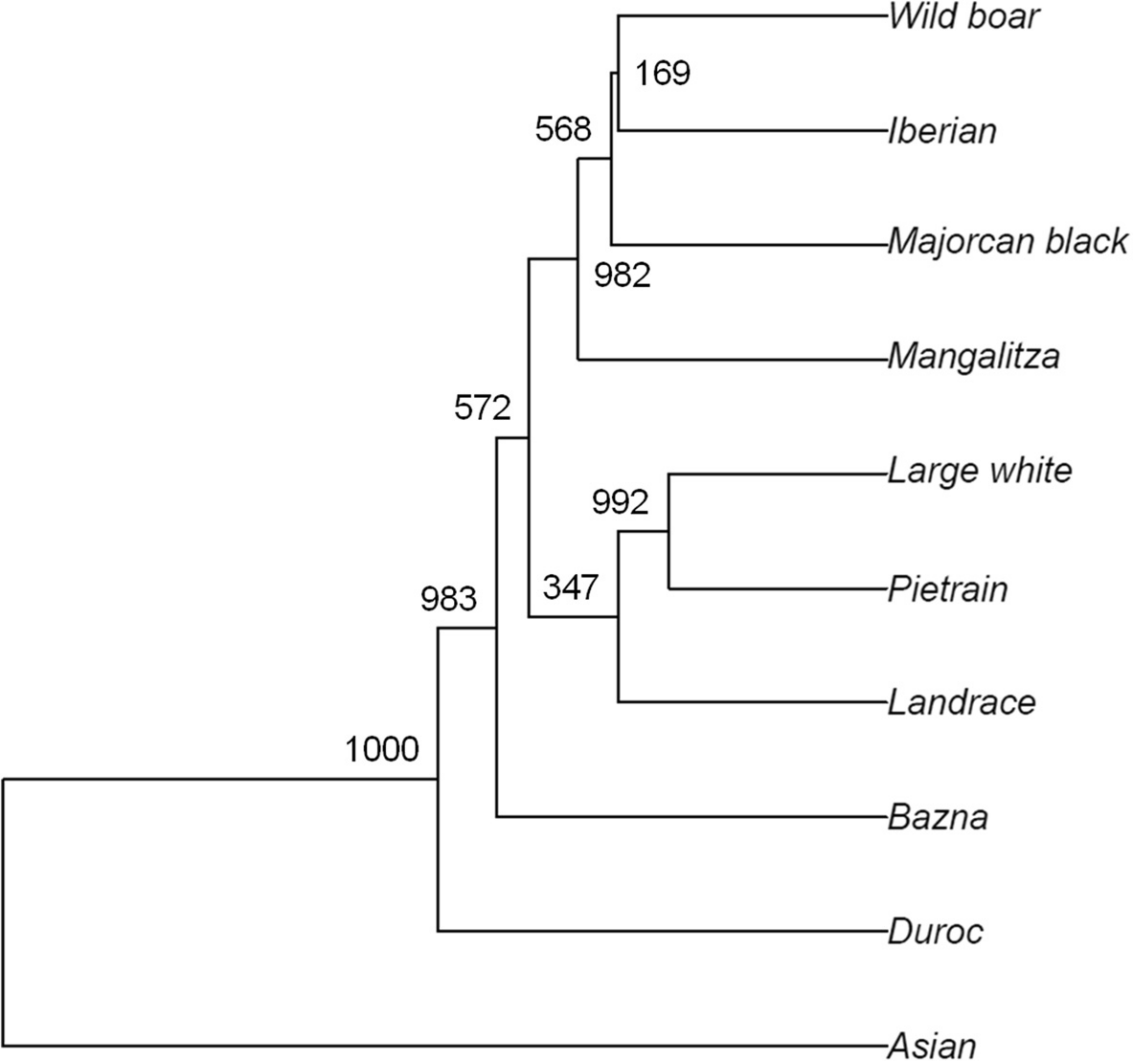
Phylogenetic dendrogram with the 10 breeds. The numbers indicate the support for each node according to 1,000 bootstrap iterations

We performed hierarchical clustering using an Unweighted Pair Group Method with Arithmetic Mean (UPGMA) based on the 2,523 (217 TASR and 2,306 AR) variants that were present in at least one of the ten breeds and with pAAF information available in all these populations. One-hundred variants were excluded from the purebred comparison since they were unique to the F_2_ animals. The resulting phylogenetic dendrogram is in line with what has been shown in other studies (Fig. 2). Briefly, the Western (European and USA) breeds cluster together and the Asian pool form a separate branch. Within the Western cluster, the Duroc is the only member of a distant branch whilst the European commercial breeds (Large White, Landrace and Pietrain) belong to another sub-group and the more ancient breeds Iberian, Majorcan Black, and Mangalitza cluster together with the wild boar.

### pAAF and phenotype relationships in the F_2_ groups

We also wanted to see whether we were able to detect an indication of an effect of the variants on production traits. This was investigated by comparing the pAAFs of two F_2_ pools from the same experimental population, each belonging to one of the tails of the phenotypic distribution, for average daily gain and retroperitoneal fat content. Out of the 217 TASR coding variants, 97 segregated in the F_2_ resource, and within these, eight displayed significantly different pAAFs in five TASRs (Table 5 and Additional file 4). Remarkably, four *TAS2R4* variants showed significant differences between the pools. Likewise, we could compare 1,280 variants in AR genes segregating in this population and identified 56 significant differences (*p-val* ≤ 0.05) involving 25 genes (Table 5 and Additional file 4). After correcting for multiple testing (p-val ≤ 0.05/(97 + 1,280) = 0.00003), one M variant in *TAS2R39* and three variants in *CD36* remained significant (Table 5).

**Table 5:** Genes with variants showing significantly different pAAF between F2_F and F2_L

Gene	Total number	Number and type of variants with significant differences is pAAF	Fisher Test value range (min-max)
*ADRB1*	1	1 synonymous	0.0467
*ALDH1B1*	3	3 synonymous	0.0253
*ALDH2*	1	1 non-synoymous tolerated	0.0358
*ALDH3B2*	7	1 non-synoymous deleterious; 6 synonymous	0.0003–0.0137
*ALDH6A1*	1	1 synonymous	0.0253
*CD36*	3	1 frame-shift; 1 non-synoymous deleterious; 1 synonymous	1.5xE-05^a^ - 2.58xE-05^a^
*DISC1*	3	3 synonymous	0.0280–0.0357
*TVPR1* human ortholog (ENSSSCG00000017863)	5	1 non-synoymous deleterious; 4 synonymous	0.0111–0.0315
*ALDH8A1* ortholog (ENSSSCG00000023457)	1	1 synonymous	0.0387
*FOS*	2	1 non-synoymous tolerated; 1 synonymous	0.0284
*GABRA3*	1	1 splice-site donor/acceptor	0.0253
*GABRA6*	1	1 synonymous	0.0047
*GPR179*	9	2 non-synoymous deleterious; 4 non-synoymous tolerated; 3 synonymous	0.0137–0.0324
*GPRC5B*	1	1 non-synoymous deleterious	0.0253
*GPRC5C*	1	1 non-synoymous tolerated	0.0258
*GRM1*	3	3 synonymous	0.0047–0.0383
*GRM8*	1	1 synonymous	0.0178
*HTR1B*	1	1 synonymous	0.0253
*HTR3C*	1	1 synonymous	0.0357
*LEPR*	1	1 synonymous	0.0253
*MCHR2*	2	2 synonymous	0.0115–0.0253
*MTNR1B*	1	1 non-synoymous tolerated	0.0357
*P2RX2*	2	2 synonymous	0.0094–0.0324
*P2X7*	3	3 synonymous	0.0006–0.0324
*SIM1*	1	1 synonymous	0.0115
*TAS2R16* ortholog (ENSSSCG00000016433)	1	1 synonymous	0.0315
*TAS2R39*	1	1 codon change	6.16xE-08^a^
*TAS2R4*	4	2 non-synoymous tolerated; 2 synonymous	0.0002–0.0158
*TAS2R41*	1	1 non-synoymous tolerated	0.0207
*TAS2R60*	1	1 synonymous	0.0351
Total	64		

^a^Variants with Fisher test values remaining significant after Bonferroni correction for the multiple testing for 1,377 variants (p-val ≤ 0.05/(1,377))

### Selection and genotype-based validation of a set of protein-damaging variants

**Fig. 3:**
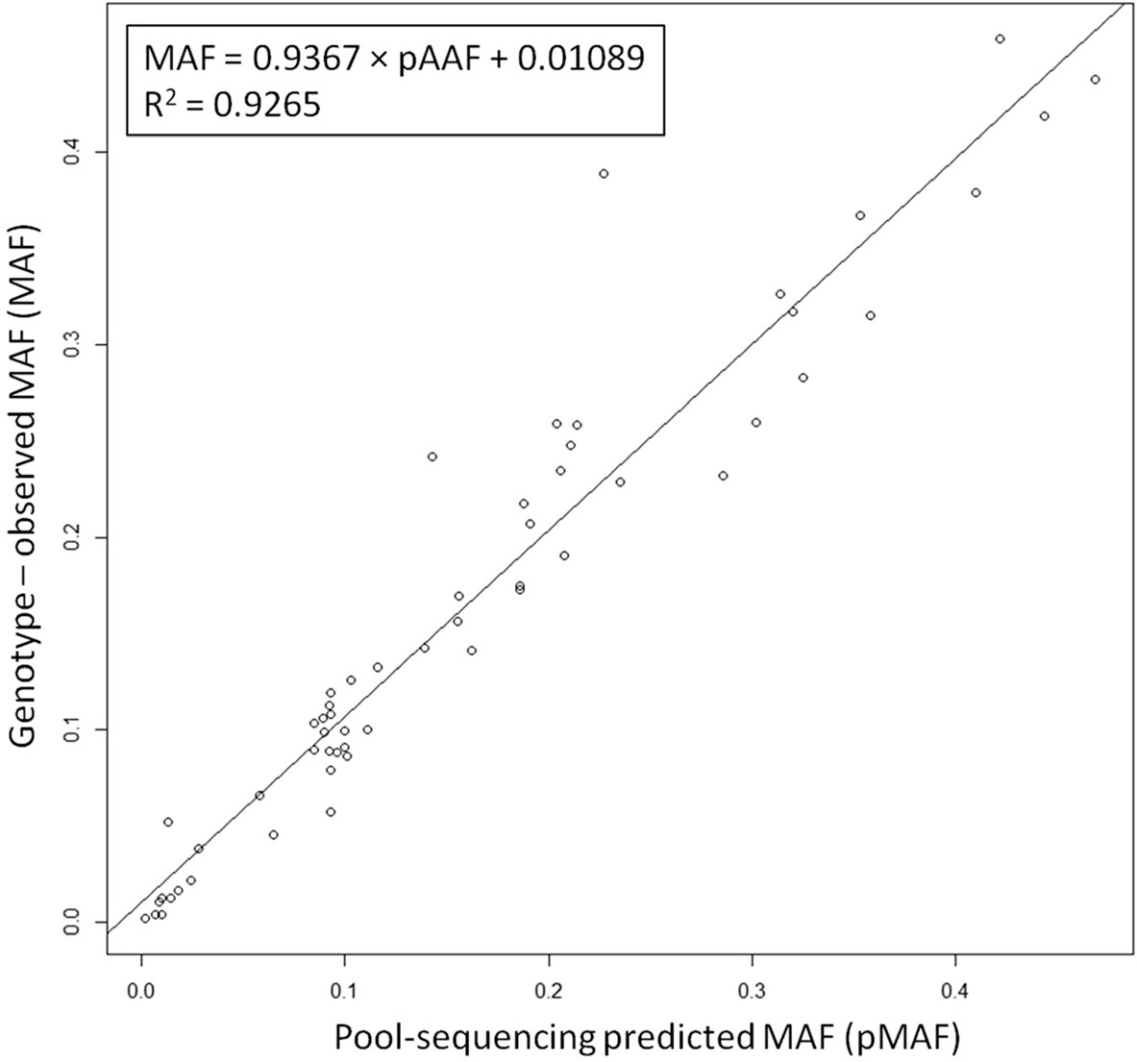
Linear regression and correlation between pMAF and the genotype observed MAF in the 57 genotyped polymorphisms

With the double aim to validate the subset of variants with more likely damaging impact and, to evaluate by genetic association, their impact in pig breeding, we designed a TaqMan genotyping assay targeting 128 putative polymorphisms. We originally aimed at including all the H variants found in the study and to fill the remaining of the assay with several M variants to cover all TASRs and several AR genes. However, the assay had design requirements that not all the variants fulfilled. Hence, we could only include nine H variants in TASR, 69 H variants in AR genes, 33 M variants in TASRs and 17 M variants in AR genes. To validate the selected variants, we genotyped 237 (Additional file 5) of the 304 sequenced pigs. We could not genotype the remaining 67 pigs as all their DNA was used for the sequencing step. 9 % of the assays failed to either amplify or clearly differentiate the genotype clusters, and 46 % did not show the alternative allele in any sample. 57 variants identified in our variant calling pipeline were validated by the TaqMan assay. The 57 polymorphisms (3 TASR H, 12 AR H, 31 TASR M and 11 AR M) affect eight TASRs and 23 AR genes (Additional file 6). All genotyped variants showed to be in Hardy-Weinberg Equilibrium across breeds (data not shown). Although none of the four genotyped rare polymorphisms (all H) showed a homozygote state for the minor allele, one H SNV (chr16_45548628_G_A), unique to the Vietnamese pigs and with a MAF slightly above the rare variant threshold (MAF = 0.013), was found in the homozygous state in two animals. This variant was predicted to cause a stop codon gain in the very last amino acid of the canonical HTR1A protein. Another introduction of a stop codon (chr8_38038074_G_A) was found in the middle of the Gamma Aminobutyric Acid receptor *GABRG1*, and was present only in Meishan pigs showing a MAF = 0.022. We next compared the predicted and the matched genotype-observed frequency of the minor allele and detected a strong correlation (r^2^ = 0.93) between the two (Fig. 3).

We also carried a genetic association analysis with the genotypes and phenotypic values for retroperitoneal fat and daily gain in the F_2_. 36 of the 57 variants segregated in the F_2_ animals. The array contains two of the variants (chr2_3566521_A_C and chr18_7019623_G_A) that showed significant pAAF differences between the F_2_ groups with the Fisher test. For daily gain, only the marker chr2_3566521_A_C was nominally significant (p-val = 0.025) (Additional file 7) but it did not reach significance after Bonferroni correction for multiple testing (p-val ≤ 0.05/36 = 0.0013). For retroperitoneal fat, two markers, chr9_46397500_A_G and chr18_7019387_A_T, were nominally significant (p-val = 0.028 and 0.044, respectively) but again, none reached statistical significance after Bonferroni correction (Additional file 7). Chr18_7019623_G_A, which was significant at the pAAF comparison between both F_2_ groups, did not reach significance for any of the two traits (p-val = 0.09 and 0.1 for daily gain and retroperitoneal fat, respectively).

## Discussion

TASRs and the components of the AR circuitry are key genes in keeping body homeostasis as they recognize chemical molecules that could be both sources of energy or threatening toxins and promote an adequate response. With the aim to characterize the coding genetic variation affecting swine TASRs and the AR circuitry genes, we sequenced 16 gDNA pools corresponding to 304 pigs from ten breeds and European wild boar and from two pools of an experimental F_2_ population with records on growth and retroperitoneal fat content. We have mapped thousands of coding region genetic variants, hundreds of which are expected to have a strong impact on protein sequence, some of which are breed specific. By comparing the pAAFs of these variants in two F_2_ pools divergent for growth and fat deposition, we also identified many genotype - phenotype relationships. Our data provides detailed information of the genetic variation present in TASRs and AR genes. We also developed an assay to genotype 128 of the most functionally relevant variants which is available to perform association studies with relevant traits in pig populations.

### Technical considerations

Differences in DNA extraction methods and inaccuracies with respect to quantification might have a negative impact on the even distribution of sequencing reads among the genomes within a pool thereby reducing the accuracy of the pAAF/pMAFs as a proxy of the real allelic frequency. Nonetheless, the comparison of predicted versus observed allele frequencies in the 57 genotyped variants showed that pAAF/pMAFs were very good predictors and supply additional confidence of the quality of our results (Fig. 3).

To the best of our knowledge, this is the first published study from a targeted genome enrichment experiment in swine. As this approach reduces the sequencing throughput requirements, we were able to sequence the target sub-genome (the exome of 201 TASRs and AR genes) in the largest number of pigs (*n* = 304) reported to date, in a single experiment. By mapping nearly 163 million reads on the target exons, we reached an average depth of sequencing of 72× for each of the haploid genomes. This read depth allowed us to detect all variants present in the pools regardless of their frequency. Given that we sequenced 304 animals harbouring in total 608 chromosome sets (2 alleles each), we were able to detect rare variants with MAF ≥ 0.0016. No previous high throughput sequencing study in pigs reached this sensitivity to detect rare variants. We identified a large number of variant events, most mapping to exon flanking regions including promoters, introns, and upstream and downstream segments. Although some of these non-coding variants could be functional, their regulatory relevance is difficult to predict and was not the subject of our study, which focused mainly, on the detection of protein-damaging coding variation.

### Variant identification

Overall, we have identified 2,793 coding variants but 27 of these are fixed in our sample set and are thus likely to be either errors in or private to the reference sequence. We annotated 217 variants segregating in the 10 canonical swine TASRs in our samples. Da Silva et al. [18] described 279 coding variants in their list of 21 taste and nutrient receptor genes after sequencing 79 pigs. However, Da Silva and co-authors included 8 genes that we did not study. These are 5 likely-paralogs of canonical TASRs and 3 genes that are not canonical TASRs but that are related to tasting fat (*GPR41*, and *GPR84*) and amino acids (*GPRC6A*). On the contrary, we included 2 canonical TASRs (*TAS2R4* and *TAS2R40*) not studied by Da Silva et al. Moreover, 4 we classified as AR genes (*GPR40*, *GPR120*, *CASR, GRM1 and GRM4*) that are shared in both studies as they are not canonical TASRs or are receptors belonging to the glutamate pathway. 138 of the 279 variants, 71 in canonical TASR and 67 in the other shared genes, are common to both studies. The difference in the catalogue of identified variants is likely to be mostly due to the fact that Da Silva et al. studied a different set of animals, some belonging to breeds we did not target (Hampshire, creole, Brazilian and Tamworth). 395 of the 2,766 “segregating” variants are predicted to have a high impact (H and Mdel) on the protein sequence of 133 genes and are thus very likely to disrupt or strongly alter their function (Table 1 and Additional file 2). Some of these variants showed allelic frequency relationship with daily gain and retro-peritoneal fat deposition in the F_2_ resource with phenotypic records (Table 5 and Additional file 4). In keeping with previous findings in human [13], we also detected that H and M variants tend to be more abundant and have higher pAAFs in TASRs than in non-sensory genes (Tables 1 and 2) which indicates that TASRs are subject to balancing selection. TASRs are comparable to the major histocompatibility complex genes (HLA in humans and SLA in pigs) as both are expressed at the surface of cells to detect particular molecules that could be hazardous for the body. Whilst HLA and SLA detect antigens to promote an immune reaction, TASRs sense chemical compounds to stimulate appropriate responses (reward, acrosomal reaction, smooth muscle contraction or immune function among others) depending on the cell type that is involved. Thus, a healthy animal population needs to be highly polymorphic in these genes in order to maximize its adaptability and survival to variable environments facing multiple threads. As expected, H and Mdel variants had on average, lower pMAFs than Mtol or L variants (Table 2), as the former are more likely to be deleterious and consequently, subject to purifying selection. This is well exemplified by the recent exome sequencing experiments that have successfully identified rare deleterious variants causing rare and severe mendelian disorders in humans [14]. Indeed, H and Mdel variants are more abundant and frequent at the 3′-end of the protein, where they are less likely to have an impact of the function of the protein (Fig. 1).

### H rare variants

Remarkably, we found 32 rare H variants which mapped to *TAS1R1*, *TAS1R3* and 20 AR genes (Table 3). These mutations are predicted to fully disrupt the function of the affected gene and might thus have an impact on taste and AR perceptions. Eight of the 20 AR genes carrying rare H variants, have been directly associated to taste, food intake, body size, diabetes or triglyceride levels. Two of them, *GRM4* and *GABRR1,* are glutamate receptors that have been associated to body size [21] and feeding behaviour [22], respectively. Natural knock-out pigs, (i.e., pigs homozygous or compound heterozygous for rare H variants) in these genes may have severe consequences in taste perception and feeding attitude. Reverse genetics experiments to study the phenotypic changes that occur in such natural knock-outs would be very informative to understand the importance of these mutations on feeding behaviour and on a broad range of other traits of relevance in animal breeding and bio-medical sciences. However, the identification of such homozygotes may require the screening of thousands of pigs. A convenient alternative would be to identify heterozygous animals and cross them to generate these natural knock-outs.

### Breed particularities

We found several variants with breed particularities that could both explain in part the population history of these breeds and be the result of genetic adaptation to the particular environment or artificial selection. Overall, the pools of commercial breeds (Large White, Landrace, Pietrain, Duroc) contained more variants than the pools of the ancient/traditional breeds (Iberian, Majorcan Black, Mangalitza, Bazna). The sequencing of the commercial breeds involved a larger number of samples and consequently, more genetic variation could be captured. This difference is possibly due also to first, population bottlenecks and a low effective population size within ancient breeds and second, to the introgression of Asian germplasm in the European commercial breeds [23, 24] (Table 4). The Duroc pool is somehow in between European commercial and traditional breeds, with a relative low number of variants (Table 4). However, this was expected as the pig reference genome sequence was obtained from a Duroc animal and is therefore expected to share more similarities with our Duroc pool. On the other hand, the Asian breeds showed the largest number of variants (Table 4) which was also expected as our pool was made of two different swine populations (15 Meishan and 7 Vietnamese pigs) and also, these animals diverged from the European counterparts around one million years ago [25]. It is worth pointing out that as we merged the Meishan and the Vietnamese pigs, we cannot have specific information for these populations. Although the comparison of both Asian breeds would have been interesting, our aim was not to study the genetic variation within these breeds but to identify a large catalogue of variants by sequencing a set of divergent populations. For this reason, and to optimise the high throughput sequencing resources, we merged all Asian animals.

Although we tried to minimise co-ancestry between samples, we do not have pedigree data and hence, we cannot exclude the possibility that the trend on the reduction in the genetic diversity observed in some breeds is due to close familiar relationships. This is particularly true for our Mangalitza samples, which come from a highly inbred population from Romania and might thus not represent the genetic diversity existing within the Mangalitza breed as a whole. To better determine genetic diversity within each breed, we compared the genetic variability in the synonymous sites, which are considered to be neutral in evolutionary terms, in our TASR and AR genes using the method described by Watterson. This method corrects the number of variants by the number of individuals that were analysed [20]. We further corrected these values by the expected number of silent sites in the sequenced cds. Genetic variability among western breeds is highly similar (0.00090–0.00131 variants per mutant site) being lowest in Duroc and highest in Pietrain. The genetic variability seems to be higher in the Asian pool (0.00182).

We also compared the relative abundance of H + Mdel variants in each breed. In contrast to the recent results by Bianco and co-authors [26] who detected a higher ratio of deleterious variants in Western than in Asian breeds, we did not identify large differences across pools. This inconsistency could be explained by the fact that we interrogated a particular set of genes whilst Bianco et al. assessed all annotated genes in the pig genome. Also, both studies screened different animals from distinct breeds. Furthermore, the two studies used different sequencing strategies. Whilst we deep-sequenced 304 pigs in a pool-based strategy, Bianco et al. performed low depth whole-genome shotgun sequencing of 128 pigs. Our approach is better suited for the identification of rare variants and this difference could have altered the catalogue of variants identified in both studies. Therefore, the comparison of the two datasets needs to be taken with caution. Of note, we observed that 25 % of the 28 variants identified in *TAS2R1* were wild boar specific (Additional file 3). This could indicate a particular haplotype that might have been lost in the European domestic breeds by artificial selection. Alternatively, as our wild boar gDNA pool was made with samples from three different European locations (Catalonia, Belgium, Romania), these variants, which have a relatively low pAAF [0.16–0.18], could well belong to one of these populations with no germplasm contributed to the analyzed domestic breeds. Noteworthy, some of these *TAS2R1* variants are predicted to have a strong effect on the protein and could thus indicate adaptive selection to particular foods or environments (Additional file 3). Especially relevant are the breed-specific H variants with relatively high frequency in the affected breed (pAAF > 0.1) (Additional file 3). The stop codon that prematurely truncates *TAS1R1* right in the middle of the protein in 17 % of the Mangalitza genomes might impair the ability to sense umami and might thus affect food preferences in a similar way as described in giant pandas, which lack a functional *TAS1R1* potentially disrupting preferences for protein-rich sources [27]. Furthermore, two H variants in *HTR3C*, and *CYP2A6* are unique and almost fixed in the Asian breeds (Additional file 3). As the pAAF between Asian and European pools are so dramatically different, we hypothesize that this might reflect an AR adaptation to very different environments.

### Variant and phenotype relationships

In our study, we aimed not only at identifying damaging variants but also at checking whether we were able to detect potential relationships with production traits. This comparison was done with only 38 pigs, which would typically be a very small number of animals and as such, the power to detect genetic associations is low. Thus, it does not aim at finding statistically significant genetic associations but at detecting a trend that could indicate this association. Indeed, we identified several allele - phenotype relationships that could indicate real genetic associations (Table 5). We believe that comparing the pAAFs of the two F_2_ pools was a good strategy for the identification of allele - phenotype relationships, as these animals share a common genetic background and the only criteria used to make the pools, was their extreme and opposed phenotypic characteristics. Four of the 11 (36 %) *TAS2R4* variants segregating in our F_2_ resource showed significant differences at the nominal level in pAAF between the obese and fast growing F2_F pool and the lean slow growing F2_L pool (Table 5). As this is a large percentage, we believe that this could be a real association and that a polymorphism, perhaps a regulatory variant not assessed here, is in part responsible of the phenotypic differences between the divergent groups of pigs. Five of the six genes with significant allelic differences at the nominal level (all with p-val ≤ 0.01) between the F_2_ pools have been directly linked to both taste and growth (umami taste and *GRM1* [28], fat taste and *CD36* [29], taste in general and *P2RX2*/*P2RX7* [30] and weight gain and *GABRA6* [31]) (Table 5) and could thus indicate a difference in the eating behaviour. This would explain the difference in growth and fat deposition between the F_2_ groups. Remarkably, three variants in *CD36*, a gene associated to fat taste, had significant pAAF differences between the high and the low fat deposition pigs after correction for multiple testing (Table 5).

Two of the markers, chr2_3566521_A_C and chr18_7019623_G_A, in the *ALDH3B1* ortholog ENSSSCG00000026349 and in *TAS2R41*, respectively, were also genotyped in the F_2_ animals with TaqMan probes using the OpenArray technology. Only chr2_3566521_A_C was nominally significant for daily gain (p-val = 0.025), but did not reach the significance threshold after multiple testing (Additional file 7). chr18_7019623_G_A was not significant, but a marker 236 bp upstream from this SNP, chr18_7019387_A_T, was nominally significant (p-val = 0.044) for retroperitoneal fat content (Additional file 7). We seek to screen larger populations with phenotypic data to determine whether these associations could become significant.

### Selection and genotype-based validation of SNPs

We also developed a genotyping array to validate the variants with highest damaging potential regardless of their frequency. We believe that a proportion of the 59 non-polymorphic positions might be real low-frequency polymorphisms that were present only in animals that we did not genotype due to the lack of available gDNA. However, we cannot rule out the possibility that some of these variants could simply be false positives caused by the erroneous mapping of some reads to highly similar regions. For example, none of the 17 frame-shift variants we included could be confirmed as a polymorphism but the high false positive rate among indels in high throughput sequencing experiments is well documented. Most of the H variants failed to proof polymorphic but they had very low pMAFs whilst most of the M variants, which on average showed higher pMAFs, were confirmed (Additional file 6). None of the rare H polymorphisms displayed the homozygous state for the minor allele in the genotyped samples. However, we identified two H variants that approached the rare MAF threshold, which showed the three genotypic classes in our animals (Additional file 6). The Vietnamese rare variant in *HTR1A* is a stop codon that was homozygous in two Vietnamese pigs. Nonetheless, we cannot consider these two animals as natural knockouts as this mutation is located at the very last amino acid of the canonical HTR1A protein, and it is not expected to have an impact on *HTR1A* function. Therefore, the low frequency of the minor allele might not reflect the existence of purifying selection forces. The Meishan H variant in *GABRG1* is also an introduction of a stop codon, but this is located in the middle of the gene and is thus likely to disrupt its function. This gene increases neuronal activity and is associated to eating disorders and anxiety [32] and even with alcohol dependence in humans. Thus, natural *GABRG1* knock-out pigs carrying this stop codon may show both signs of anxiety and a particular eating behaviour.

We have developed the first assay for massive genotyping of variants with high functional potential in swine TASR and AR genes, and we now seek to perform association analysis on pig populations with phenotypic records for eating attitude, feed intake, growth, obesity, but also on semen quality and fertility, infection and immunity and behaviour abnormalities such as stress-related stereotypies and tail biting. This approach will help us understanding the impact of genetic variation in TASR and AR genes on traits of interest for the pig breeding industry. This genotyping assay will also allow us to identify natural knock-outs that could then be used in reverse genetics studies. Nonetheless, we acknowledge that this array tags a very small proportion (eight TASRs and 23 reward) of the genes involved in taste, appetite and reward. Ideally, the list of variants and genes should be extended to achieve a comprehensive analysis of the impact of these gene pathways in pig breeding.

## Conclusions

We detected 2,766 variants predicted to have a potential (high, moderate or low) impact in the protein sequence of 201 TASR and AR genes. Of these, 395 were predicted to strongly impact on the protein sequence of the 10 TASRs and 123 AR genes and consequently, in their function. The importance of these genes in many traits contributing to body homeostasis has been well documented in human and animal models but remains unexplored in livestock. We have found significant relationships between the pAAF of some variants and growth and fat deposition. This, albeit not more than a mere indication, strongly encourages further studying the effect of these genes on traits of interest in body homeostasis and animal breeding. For this reason, we have developed a genotyping array with a subset of these variants and have validated 57 by genotyping the initially sequenced animals. This array is now ready to be used in genetic association studies for relevant traits including taste preferences, food intake, fertility or behaviour. Although this array is not comprehensive and does not contain all the variants that we identified, it contains a careful selection of the most likely deleterious variants and involves eight TASRs and 23 AR genes.

## Methods

### Selection of target regions

The genomic regions of TASR exons were selected using the genome annotations accessible via Ensembl’s Biomart (www.ensembl.org/biomart; version 72, June 2013). We selected two TAS1Rs and the 10 annotated TAS2R genes (Additional file 1). *TAS1R2* could not be included due to its unknown genomic location at the time of selection.

In total, 166 genes from the AR circuitries were selected from a study aimed at understanding the genetic signatures in giant panda that confer the highly selective diet based on bamboo only [33]. In their study, these authors retrieved the 166 genes from 4 review articles on appetite and food intake behaviour as described in their material and methods. In addition, 35 AR genes were identified by searching NCBI’s PubMed database using the keywords: “appetite”, “food intake”, “dopamine”, “serotonin”, “glutamate receptor”, “epinephrine”, “norepinephrine”, “reward”. We used Ensembl’s Biomart to identify and select the genomic coordinates of the exons from the swine AR orthologous genes (Additional file 1).

### Samples

We used gDNA from 266 pigs belonging to eleven breeds or populations including Large White (United Kingdom), Landrace (Denmark), Pietrain (Belgium), Duroc (United States), Iberian (mainland Spain and Portugal), Majorcan Black (Balearic islands), Bazna (Romania), Mangalitza (Hungary), Meishan (China), Vietnamese pot-bellied and to the European wild boar. The Large White, Landrace, Pietrain and Duroc have been subjected to strong selection pressures in the last decades and at present are the most commonly used in intensive production systems. On the contrary, Iberian (mainland Spain and Portugal), Majorcan Black (Balearic islands), Mangalitza (Hungary, although our animals come from a closed herd in Romania) and Bazna (a Romanian breed obtained in the 19th century by crossing Mangalitza and Berkshire) are local breeds with much lower selection intensity and with very localised geographic locations and relatively small productions. The Large White was strongly introgressed with pigs from Asian origin back in the 18th and 19th century. The Landrace was developed in the late 19th century by crossing native Danish breeds with Large White pigs. All samples were collected from farms in Catalonia, Canada and France. Samples were selected based on DNA availability in our DNA archive. Although we do not have pedigree information, we tried to minimise co-ancestry by selecting samples from different farms when possible with the exception of the Mangalitza, which samples come from a close and highly inbred population and are thus likely to have strong familiar relationships. No phenotypic data is available from these samples. Furthermore, DNA from 38 F_2_ pigs from an experimental intercross created with the aim of studying obesity-related traits was used. This experimental intercross is described in detail by Kogelman et al. [34]. Briefly, the F_2_ population was created by inseminating seven Large White and seven Duroc sows with Göttingen minipig semen from 14 males. Both Large White and Duroc breeds have been selected for leanness and growth traits during many years, while Göttingen minipigs are prone to obesity. 563 F_2_ pigs were created. The animals were housed at a regular pig farm, and slaughtered at a commercial slaughterhouse under veterinary supervision. Tissue and blood samples were collected at slaughter. Extensive phenotypic collection was performed from birth to slaughter (242 ± 48 days) including obesity, obesity-related, and metabolic phenotypes; and measurements of fat compartments at slaughter [34]. The retroperitoneal fat compartment was removed from the carcass by blunt dissection and weighted on a bench scale. To calculate daily gain, body weight was measured individually at birth and at 7 month of age (220 ± 45 days). The 38 F_2_ pigs used in this study were selected to represent two divergent groups with respect to average daily gain (g/day) and retroperitoneal fat content (kg). The two divergent groups, i.e. the fast growing, obese (F2_F) and the slow growing, lean (F2_L), showed highly significant differences (*T*-test) for both traits (Additional file 8). gDNAs from purebred animals were extracted using different tissues (blood and a variety of solid tissues) and protocols including standard phenol - chloroform - Isoamyl alcohool organic extraction and the Charge Switch gDNA Micro Tissue kit (Invitrogen). DNA from the F_2_ pigs was extracted from EDTA stabilized blood using a salting out procedure. Samples were pooled in 16 tubes on a per-breed basis using semi-equal amounts of gDNA as measured by Nanodrop. The sample size of the pools ranged between 12 and 24 (Additional file 5). Likewise, the sample size of each breed ranged between 12 and 45 (Additional file 5).

The F_2_ animals belong to an experimental population in Denmark and they were subject to animal care, maintenance and experimental work according to the ‘Animal Maintenance Act’ (Act 432 dated 09/06/2004) and the approval from the Danish Animal Experimentation Board (J.nr. 2007/561-1434). Specialized professionals at each institution obtained all the other blood and tissue samples following standard routine monitoring procedures and relevant guidelines. No animal experiment has been performed in the scope of this research.

### Capture of genomic regions, library prep and high throughput sequencing

The 16 gDNAs pools were subjected to genomic capture and library preparation following Agilent’s SureSelect protocol for Illumina paired-end sequencing. Briefly, three μg of porcine gDNA were sheared on a Covaris™ E220 instrument. The fragment size (150–300 bp) and the quantity were confirmed with the Agilent 2100 Bioanalyzer 1000 chip. Fragmented DNA was end-repaired, adenylated and ligated to Agilent specific paired-end adapters. The DNA with adapter-modified ends was PCR amplified (six cycles, Herculase II fusion DNA polymerase). PCR product size and quantity were determined on the Agilent 2100 Bioanalyzer DNA 1000 assay and hybridized to the genomic capture baits for 24 h at 65 °C (Applied Biosystems 2720 Thermal Cycler). The hybridization mix was washed in the presence of magnetic beads (Dynabeads MyOne Streptavidin T1, Life Technologies) and the eluate was PCR amplified (16 cycles) in order to add the indexed tags using 6 bp SureSelectXT indexes for Illumina. The final library size and concentration was determined on an Agilent 2100 Bioanalyzer 1000 assay.

Each library was sequenced on an Illumina HiSeq 2000 instrument in a fraction of a sequencing lane following the manufacturer’s protocol, with paired end run of 2x101bp. Images analysis, base calling and quality scoring of the run were processed using the manufacturer’s software Real Time Analysis (RTA 1.13.48) and followed by generation of FASTQ sequence files by Illumina’s proprietary CASAVA software.

### Read mapping and variant calling

Reads were hard trimmed from the end of the read up to the first base with a quality of at least 10. Reads with at least 40 nt of length were mapped to *Sus scrofa* reference version 3 (http://hgdownload.cse.ucsc.edu/goldenPath/susScr3/bigZips/susScr3.fa.gz). As the list of TASRs was clearly shorter than the catalogue of AR genes and to minimize type I error (false negative variants), we applied a slightly different read mapping strategy for the two gene sets: 1) For TASR variant detection, reads were mapped first with the GEM toolkit [35] allowing up to four mismatches, and unmapped reads were then aligned to the swine genome using the more permissive BFAST read aligner [36]. We further manually curated the TASR variant list by removing these variants that clustered in high variant density regions as these indicate the presence of wrongly mapped reads - most of them probably aligned by Bfast - and thus false variant calls; 2) For the detection of variants in the AR genes, reads were mapped using only GEM as the manual curation would have been too labour intensive and prone to errors. Alignment (.bam) files containing only properly paired, uniquely mapped reads without duplicates were submitted to variant calling. Each pool was processed separately. The ploidy of the pool was calculated as two times the number of individuals in the pool and input as an optional argument in GATK 3.1 UnifiedGenotyper [37]. For variant calling, read numbers were down-sampled to 1,000 reads per position.

Single pool variant calls were merged into a multi-sample.vcf file using GATK CombineVariants [37]. To confirm that variant positions not called in certain pools had a homozygous reference genotype, a second round of single pool variant calling was performed restricted to the list of variant positions in the merged.vcf file. Subsequently, results were merged again. Functional annotations were added using snpEff [38] with the Sscrofa10.2.69 database, and variants were classified according to their predicted impact as High (H), Moderate (M), Low (L) and Modifier (Additional file 9). Porcine dbSNP version 138 and porcine SIFT scores and deleteriousness prediction were annotated using snpSift [39] and the Ensembl Variant Effect Predictor (VEP) online tool (http://www.ensembl.org/Tools/VEP). M variants were further classified as deleterious (Mdel) or tolerated (Mtol) according to SIFT predictions. Genes of interest and the original target region of the capture experiment were annotated using vcftools [40]. Base counts at variant positions in the merged.vcf file were annotated using GATK Variant Annotator [37]. We used the proportion of reads carrying each allele as an estimator of the allelic frequency for both the alternative allele (pAAF) and for the minor allele (pMAF).

### Identification of breed-specific variants

We searched for allelic variants that were uniquely present or uniquely absent in only one breed. We chose these variants that were either breed-specific but with a pAAF in the specific breed ≥ 0.1 and the variants that having a pAAF ≥ 0.5 in all breeds, were absent in only one breed. These breed specific features were assessed on the 2,793 variants identified in TASR and AR genes.

### Phylogenetic tree

We calculated the pair-wise Pearson correlation of the pAAFs from the eight European breeds, the wild boars and the Asian animals (Meishan and Vietnamese together) with the 2,523 variants with the alternative allele present in at least one breed and with allelic information in the ten breeds and the wild boar. We then constructed a phylogenetic tree using an UPGMA method. These calculations were done using an in-house developed R script (Do.upgma.pops.bootstrap; https://bioinformatics.cragenomica.es/numgenomics//people/sebas/software/software.html).

### Assessment of allele frequency – phenotype relationships

pAAFs at each variant position between the two F_2_ pools F2_F and F2_L were compared and the significance of these differences were determined using the Fisher exact test in an R environment. We excluded multi-allelic variants and variants with no read count in at least one pool from the analysis. After this filtering, we were able to compare 1,377 variants.

### OpenArray design, genotyping and variant validation

In order to validate the most likely deleterious variants and to develop a genotyping assay to be used in future association studies in pig populations with relevant phenotypes, we developed a genotyping array containing 128 of such potential polymorphisms. We chose the TaqMan® OpenArray® Real-Time technology for Genotyping (Life Technologies) and designed the baits using the online Custom Assay Design tool. This design requires the absence of polymorphisms in the 2 bp window centred at the target variant and has also some constrains on the probe’s melting temperature. As a consequence, we could not design assays for all the variants using this approach. We first selected all the H variants in TASRs and AR genes and supplemented the assay with M variants in both gene groups.

The genotyping was performed in a QuantStudioTM 12 K Flex Real-Time PCR System (Life Technologies). This platform is a high performance, high-throughput technology based on real-time PCR, which enables to run up to 12,000 data points, including SNV, small insertions and deletions (indels), simultaneously.

250 ng of gDNA and master mix were loaded to the OpenArray plates using the AccuFillTM robotic system (Life Technologies), filled with an immersion fluid and sealed. OpenArray plates were genotyped according to the manufacturer’s recommendations. Genotype analysis was performed using both Taqman Genotyper version 1.3 and Symphoni Suite software (Life Technologies).

### Genetic association of OpenArray genotypes with daily gain and retroperitoneal fat in the F_2_

We used a univariate mixed model to determine genetic associations of the 36 polymorphisms genotyped and segregating in the F_2_ animals with the phenotypic records for daily gain weight and retroperitoneal fat content. The analysis was calculated with the software GEMMA [41].

## Abbreviations

AR, appetite and reward; DP, read depth at the position; gDNA, genomic DNA; H, high impact variants; L, variant with no apparent impact; M, moderate impact variant; Mdel, moderate deleterious variant; Mtol, moderate tolerated variant; pAAF, predicted alternative allele frequency; pMAF, predicted minor allele frequency; SNV, single nucleotide variation; TASR, taste receptor; UPGMA, unweighted pair group method with arithmetic mean

## Additional files

Additional file 1: List of successfully sequenced TASR and AR genes. Successfully sequenced genes include these genes fully or partially sequenced at a DP > 1,000 considering all the libraries together. (XLSX 25 kb) Additional file 2: List of variants identified in TASR and AR genes. This table contains all the variants, including the multi-allelic with more than one effect on protein sequence and these which alternative allele was fixed in all the animals. (XLSX 302 kb) Additional file 3: List of breed-specific variants. This table contains these variants that were present in a single breed at pAAF ≥ 0.1 or present in all breeds at pAAF ≥ 0.5 but absent in one single population. (XLSX 154 kb) Additional file 4: List of variants displaying significant differences in pAAF between F2_F and F2_L. (XLSX 145 kb) Additional file 5: Breed and sample size information for the 16 gDNA pools. Large White, Landrace, Duroc and Pietrain were sequenced in two pools according to their commercial origin. In the pool size column, the number between brackets indicates the sample size of each pool. The cells in the column ‘number of genotyped pigs’ indicate the number of pigs that were successfully genotyped for all or some of the variants included in the OpenArray. (XLSX 15 kb) Additional file 6: List of variants confirmed to be real polymorphisms by genotyping. MAF = observed minor allele frequency; * H variants which MAF is close to that of rare variants and display the three genotypic classes in the genotyped animals. (XLSX 12 kb) Additional file 7: Results on the genetic association of 36 polymorphisms with daily gain and retroperitoneal fat content in the 38 F_2_ pigs. (XLSX 13 kb) Additional file 8: Phenotype mean and standard deviation for F2_F and F2_L and the *T*-test p-value for both daily gain and retroperitoneal fat. (XLSX 16 kb) Additional file 9: Variant impact and effect classification according to SNPeff. This table describes the types of genetic variants according to their effect on protein sequence. This table has been modified from the SNPeff manual (http://snpeff.sourceforge.net/SnpEff_manual.html#input). (XLSX 11 kb)
